# Does it mean more positive or more negative? A study on central attitudes toward homosexuality

**DOI:** 10.3389/fpsyg.2025.1635208

**Published:** 2025-09-25

**Authors:** Lingfeng Guo, Shixin Fang, Yanzhe Zhang, Xuelu Li, Yu Yang

**Affiliations:** ^1^Flight Technology College, Civil Aviation Flight University of China, Guanghan, China; ^2^Department of Human Development and Family Studies, The Pennsylvania State University, University Park, PA, United States; ^3^Flight Attendant Academy, Civil Aviation Flight University of China, Guanghan, China

**Keywords:** attitudes toward homosexuality, central components, attitudinal measurement, attitudinal inference, complex network analysis

## Abstract

**Objectives:**

Attitudes toward homosexuality have a significant impact on the well-being of sexual minority populations. Although prior research has identified the multidimensional nature of attitudes toward homosexuality, the central components within the attitudinal structures remain underexplored. This study aimed to examine the central attitudes toward homosexuality from the perspective of both attitudinal measurement and attitudinal inference.

**Methods:**

The current study comprises two complementary studies. By assessing 666 participants’ attitudes toward homosexuality, study 1 utilized complex network analysis to identify central components within the overall attitudinal network. Study 2 adopted an attitudinal inference design to further investigate the central factors of attitudes toward homosexuality, using importance and representativeness as key indicators of centrality. Paired-sample *t*-tests and two-way ANOVA were conducted to investigate the central factor in inferring negative and positive attitudes toward homosexuality.

**Results:**

Our results of study 1 showed that the component reflecting behavioral tendencies related to social interactions is the most central in the attitudinal network. Findings of study 2 revealed that *prejudice against homosexuality* factor was more central than *preference for homosexuality* factor when inferring negative attitudes, whereas *preference for heterosexuality* factor was more central than *prejudice against homosexuality* factor when inferring positive attitudes.

**Conclusion:**

This study advances our understanding of attitudinal centrality in measuring and inferring attitudes toward homosexuality, which can offer nuanced intervention targets to reduce homonegativity.

## 1 Introduction

Attitudes toward homosexuality profoundly influence the well-being of sexual minority individuals (Huang et al., 2020). Extensive research has demonstrated that negative attitudes toward homosexuality, both externalized and internalized, can severely harm the mental health of sexual minority individuals, leading to increased levels of depression, anxiety, and psychological distress (Camp et al., 2020; Russell et al., 2011, 2012; Ventriglio et al., 2021). These negative attitudes are also associated with higher rates of behavioral issues, such as substance abuse and suicidality (Birkett et al., 2009; Espelage et al., 2008; Rosario et al., 2001). In contrast, positive attitudes toward homosexuality and the related consequences have received comparatively less attention.

Positive and negative are the most commonly used categories for describing and classifying people’s attitudes toward homosexuality. Compared to positive attitudes, people are generally more inclined to perceive and infer negative attitudes (Carretié et al., 2001; Rozin and Royzman, 2001). Existing theories on attitudes toward homosexuality predominantly emphasize the negative aspects, such as homophobia (Weinberg, 1972), old-fashioned homonegativity (Herek, 1988), and modern homonegativity (Morrison and Morrison, 2003), yet largely overlook the positive aspects. This emphasis may stem from historically entrenched views of homosexuality as pathological, sinful, or immoral within various sociocultural contexts (Herek, 1984).

Examining the centrality of these components offers both theoretical and practical contributions. Theoretically, it provides a more comprehensive understanding of the structural properties of attitudes toward homosexuality. Practically, identifying the most central components can inform the development of targeted intervention programs aimed at reducing prejudice against sexual minority populations.

### 1.1 Theories and measurements of attitudes toward homosexuality

Homophobia is one of the earliest theoretical frameworks developed to describe hostile reactions and irrational fears toward sexual minority individuals (Weinberg, 1972). From an essentialist perspective, homophobia is thought to have evolutionary significance (Neuberg et al., 2011), as homosexual behavior is perceived to contradict the fundamental reproductive purpose of human sexuality (James, 1890; Westermarck, 1908).

As research on attitudes toward homosexuality advanced, the term “homophobia” has become increasingly controversial in both academic and clinical contexts. Theoretically, homophobia conceptualizes negative attitudes toward homosexuality as a pathological condition and an inherent trait. This perspective frames individuals with homophobic attitudes as victims of a psychological condition rather than perpetrators of harm against sexual minority individuals, thereby legitimizing homophobia as an immutable characteristic (Lyonga, 2019). Furthermore, empirical evidence also challenges the assumptions of homophobia theory. For instance, research did not support the behavioral immune system hypothesis, which suggested that heterosexual individuals’ hostile attitudes toward gay men were driven by pathogen disgust; instead, these attitudes were found to be associated with sexual disgust (Ray and Parkhill, 2021). In sum, there is growing recognition that negative attitudes toward homosexuality are not innate traits but are more likely shaped by sociocultural factors related to sexuality (Herek, 2007; Russell and Bohan, 2006).

The theories of old-fashioned and modern homonegativity offer a sociocultural framework for understanding negative attitudes toward homosexuality. Larsen et al. (1980) argued that prejudice against homosexuality shares similarities with racial and gender prejudice. Old-fashioned homonegativity is defined as a negative attitude grounded in traditional religious and moral beliefs about homosexuality. It is typically measured through various manifestations, including condemnatory and demeaning cognitive evaluations, intense negative emotional reactions, and aggressive behavioral tendencies toward sexual minority individuals. Some aspects of these measures overlap with those associated with homophobia, such as the pathologization and moral condemnation of homosexuality.

Modern homonegativity theory posits that prejudice against homosexuality in contemporary society has evolved from old-fashioned homonegativity, characterized by overt expressions such as violence, aggression, and demeaning attitudes, to more subtle, moderate, and covert manifestations (Morrison and Morrison, 2003). Measurements based on this theory have been developed and applied in research and practice (Górska et al., 2017; Morrison et al., 2005, 2009). However, empirical evidence suggests that individuals can simultaneously harbor both old-fashioned and modern homonegativity, challenging the assumption that these are two mutually exclusive forms of prejudice (Lottes and Grollman, 2010). Moreover, attitudes toward homosexuality extend beyond a positive–negative dichotomy, as research has documented the existence of ambivalent attitudes that simultaneously encompass both positive and negative elements (Clausell and Fiske, 2005; Hoffarth and Hodson, 2014). Of particular note is the identification of a distinct category of discriminatorily positive attitudes (Guo et al., 2023). Modern homonegativity theory and its associated measurements, however, demonstrate limitations in evaluating these ambivalent attitudes and inadequately differentiating between purely positive attitudes and discriminatorily positive ones.

**Figure 1:**
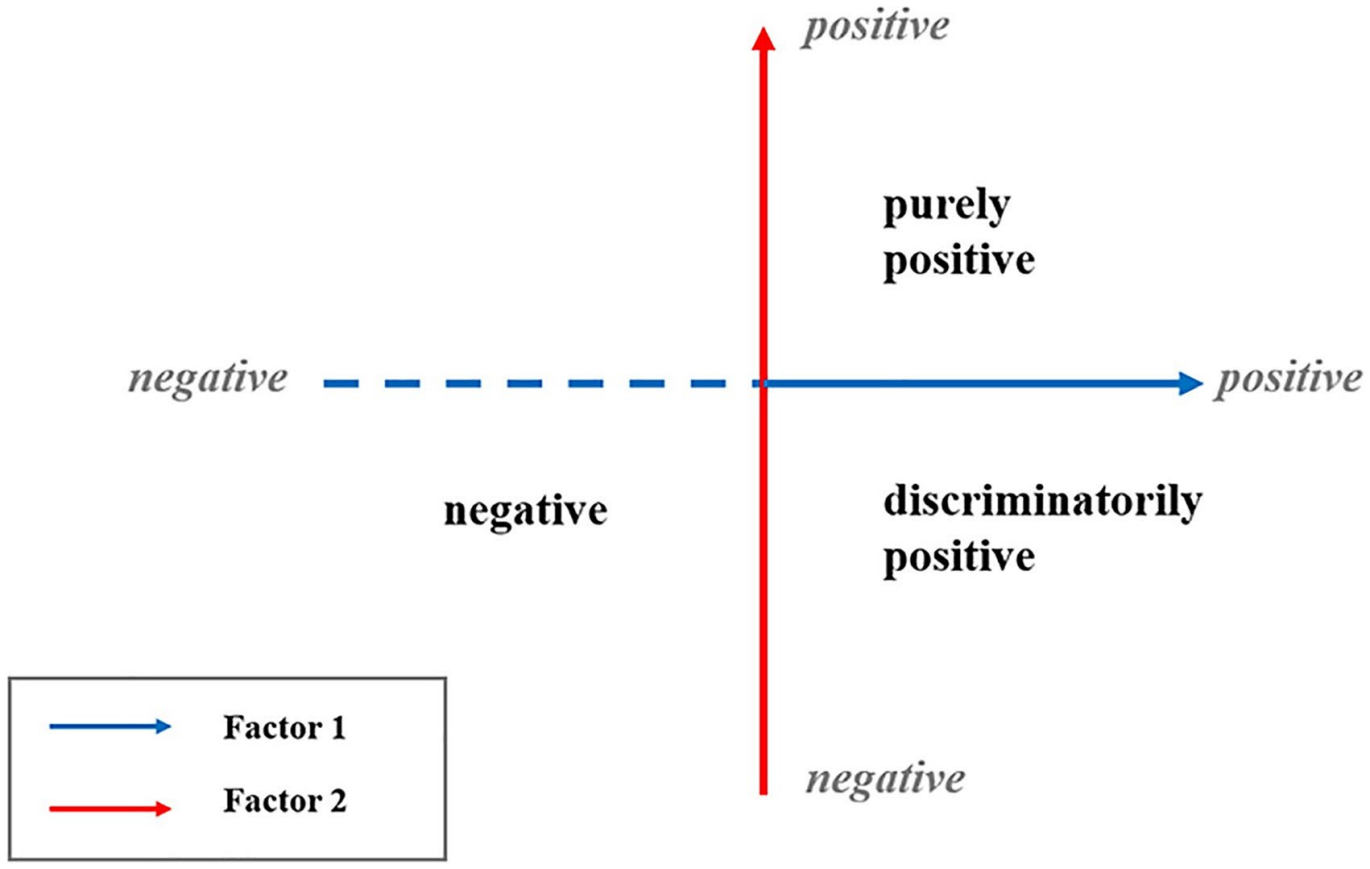
The two-factor and three-class model of attitudes toward homosexuality (Guo et al., 2023).

Guo et al. (2023) argued that negative attitudes toward homosexuality are not confined to overt aggression and hostility but can also manifest through individuals’ conscious or unconscious admiration for and reinforcement of heteronormativity. For example, politically liberal individuals in the United States often express personal support for LGBT rights, yet many also tolerate others’ prejudicial views and behaviors—thereby legitimizing others’ sexual stigma and maintaining the perceived superiority of heterosexuality (Folberg et al., 2025). In this vein, Guo et al. (2023) proposed a two-factor and three-class model of attitudes toward homosexuality and validated its measurement structure with both person-centered and variable-centered analytical approaches. The model comprises two factors: prejudice against homosexuality (Factor 1) and preference for heterosexuality (Factor 2). The combination of these two factors yielded three classes of attitudes toward homosexuality: purely positive, discriminatorily positive, and negative attitude (see Figure 1).

1. Low level of Factor 1 (95% CI [6,10]) + Low level of Factor 2 (95% CI [6,10]) = purely positive attitude.
2. Low level of Factor 1 (95% CI [6,14]) + High level of Factor 2 (95% CI [14,20]) = discriminatorily positive attitude.
3. High level of Factor 1 (95% CI [19,24]) + High level of Factor 2 (95% CI [13,22]) = negative attitude.

The two-factor and three-class model of attitudes toward homosexuality overcame the limitations of unidimensional models in capturing ambivalent attitudes toward homosexuality and challenged the positive–negative dichotomy of attitudes. Built on these initial efforts, a more comprehensive understanding of the structural properties of attitudes toward homosexuality hinges on examining the centrality of different factors and components.

### 1.2 Central components of attitudes toward homosexuality

Existing research provides evidence for a hierarchy of centrality among the components of attitudes toward homosexuality. Regarding the outward expressions of positive attitudes, research found that individuals who reported the willingness to maintain friends did not always express the same willingness to attend school with them (Paul Poteat et al., 2009). Additionally, the degree of acceptance toward homosexuality varies depending on the closeness of the relationship, such that individuals who could accept having a gay friend were not necessarily accepting of having a gay child (Liang, 2015; Tan et al., 2012). These findings suggest that under heteronormative hegemony, expressing some positive attitudes toward homosexuality face greater sociocultural pressure than others (Anderson, 2008; Duran et al., 2007; Flanders and Hatfield, 2014; Mize and Manago, 2018). Positive attitudes that require greater resistance to heteronormative norms may better represent and predict overall positive attitudes toward homosexuality, occupying a more central position within the network of positive attitude components.

The expression of negative attitudes toward homosexuality also varies in centrality. Research has shown that sexual disgust toward gay men is a stronger predictor of hostile attitudes than pathogen or moral disgust (Ray and Parkhill, 2021). Lyonga (2019) categorized negative attitudes toward homosexuality into seven forms—radical, prohibitionist, denialist, avoidance, morbidity, tepid, and veiled—each differing in intensity and manifestations. Guo et al. (2023) further examined the structure of attitudes toward gay men and lesbians and highlighted that discriminatorily positive attitudes often convey a degree of subjective goodwill despite its negative impact. Through the lens of system justification theory, such attitudes may serve as a strategy for the dominant group to sustain hierarchical power structures, which makes sexual minority individuals accept and even uphold their subordinate status (Jost and Kay, 2005; Jackman, 1994). Thus, compared to negative attitudes, discriminatorily positive attitudes can be more readily adopted by the majority group and accepted by attitude objects. Therefore, expressing negative attitudes toward homosexuality may require overcoming greater social pressure than discriminatorily positive attitudes, positioning them as more central within the network of negative attitude components.

### 1.3 Evaluating central components of attitude toward homosexuality: attitudinal measurement and attitudinal inference

Attitudinal measurement refers to the assessment of an attitude holder’s (agent) evaluation toward an attitude object (target). In the current study, it specifically denotes measuring participants’ (agents) attitudes toward homosexuality (target). Existing studies on the measurement of attitudes toward homosexuality have predominantly employed simple linear models (e.g., factor models), in which attitudes are treated as latent variables indicated by multiple observed responses to attitude items (Herek, 1988; Morrison and Morrison, 2003; Song et al., 2013). Factor loadings derived from these models can partially reflect the centrality of observed indicators. Although factor models capture the basic correlational structure of attitude components based on their shared influence by latent variables, they fall short in representing attitudes as a dynamic and holistic network (Dalege et al., 2016). To address this limitation, Dalege et al. (2016) applied a complex network analysis method to the study of work attitudes, constructing the Causal Attitude Network (CAN) model, which conceptualizes attitudes as dynamic networks of affective, cognitive, and behavioral reactions and interactions of these reactions rather than as latent variables.

The complex network analysis method constructs a network model through a series of nodes and the edges connecting these nodes. Each node represents a single attitude item response, and nodes that are strongly connected are positioned closer to one another. Nodes that are directly or indirectly connected to many others tend to occupy central positions in the network. Edges represent the partial correlations between item responses, with positive correlations depicted as green edges and negative correlations as red edges. The width and saturation of an edge reflect the strength of the partial correlation, with wider and more saturated edges indicating stronger connections (Epskamp et al., 2012). In an attitude network, connectivity represents attitude strength; greater connectivity suggests that the network is more resistant to change (Carter et al., 2020). Notably, network models share many similarities with latent variable models, and these two approaches are viewed as complementary rather than competing frameworks (Carter et al., 2020).

In the complex network model, networks are believed to exhibit a small-world structure, where similar nodes form highly connected clusters, and these clusters are interconnected to create the small-world structure (Watts and Strogatz, 1998). Networks with a small-world structure are characterized by high clustering and high global connectivity. Particularly in an attitude network, connectivity represents attitude strength and greater connectivity suggests that the network is more resistant to change (Carter et al., 2020). When one node in such a network changes, often due to targeted intervention, other nodes are also likely to be affected. This structure allows information to spread quickly within the cluster, providing a foundation for investigating the extent to which changes in one node influence others (i.e., node centrality). To determine whether a network exhibits small-world structure, the following criteria are typically used: (1) the small-world index (*SW*) is greater than one; (2) the average length of the shortest paths in the network (*L*) is either within the 95% confidence interval of a random network of the same size (*L*_rand_) or exceeds its upper bound; and (3) the clustering coefficient (*C*) is greater than the upper bound of confidence interval of *C*_rand_^1^ (Carter et al., 2020).

Centrality of a given node refers to its structural importance within a network, determined by its position and the number and strength of edges connecting it to other nodes (Freeman, 2002; Opsahl et al., 2010). Four common metrics are used to quantify node centrality (Carter et al., 2020; Dalege et al., 2016): (1) *Strength* represents the overall extent of a node’s connections with other nodes in the network; (2) *Expected influence* is an adjusted metric derived from *strength*, accounting for the inability of *strength* to aggregate negatively correlated edges adequately; (3) *Closeness* reflects the weighted sum of the distances between a node and all other nodes in the network; and (4) *Betweenness* measures whether a node lies in the shortest path between two other nodes, and changes to a node with high betweenness are likely to spread through the entire network. The complex network model has been applied to attitudinal research in areas such as job satisfaction (Carter et al., 2020) and science interest (Sachisthal et al., 2019). In this vein, this study aims to leverage complex network model to investigate the centrality of components within the holistic network of attitudes toward homosexuality.

Attitudinal measurement is typically conducted using systematic and validated scales to obtain evaluative reactions. Based on such data, complex network analysis can be employed to identify the central components of attitudes toward homosexuality and to examine the dynamic, non-linear relationships among these components. Such an investigation emphasizes understanding the structural properties and theoretical framework of attitudes toward homosexuality.

In contrast to self-reported attitudinal measurement, attitudinal inference refers to the process in which participants adopt a third-party perspective to infer an agent’s attitude toward a target (e.g., homosexuality). Participants’ attitudinal inference relies on limited and specific behaviors, using both deductive and inductive reasoning to evaluate the relationship between others’ attitudes and their external expressions. The investigation based on attitudinal inference focuses on identifying which components are most effective in determining the type of attitude toward homosexuality (e.g., positive or negative). In attitudinal inference research, the representativeness and importance of a component are often used as indicators of its centrality (Jiao et al., 2022). Representativeness refers to the extent to which a behavior or expression reflects positive or negative attitudes, while importance denotes the significance of a behavior or expression in the inference process. Importance is typically evaluated using forced-choice methods to distinguish essential components from supplementary ones (Cottrell et al., 2007).

Research on personality traits highlights that the criteria for evaluating good and evil personalities often differ (Jiao et al., 2022), and similar principles may apply to inferring positive and negative attitudes toward homosexuality. Although the absence of a specific negative expression does not necessarily imply the absence of other forms of negative attitudes (Lyonga, 2019), the presence of even a single negative expression is often sufficient to infer a negative attitude. In contrast, confirming a positive attribute is more difficult, as it requires more explicit evidence to substantiate the inference (Klein and O’Brien, 2016). Thus, this study sought to explore the central factors underlying the inference of positive and negative attitudes, respectively.

In summary, different components of attitudes toward homosexuality may vary in their centrality, and the central factors to infer positive and negative attitudes toward homosexuality may also differ. However, no existing research has systematically examined these differences in centrality across components of attitudes toward homosexuality. Therefore, this study aimed to identify central components of attitudes toward homosexuality from both attitudinal measurement and inference perspectives. By doing so, this research sought to identify the most effective intervening points within the attitude network and uncover the relationship between attitudes toward homosexuality and specific expressions, ultimately contributing to improving public attitudes and enhancing the well-being of sexual minority groups.

### 1.4 The current study

The current study employed attitudinal measurement and attitudinal inference design to identify the central components of attitudes toward homosexuality. Within the attitudinal measurement framework, Study 1 applied complex network analysis to examine the intricate relationships among components of attitudes toward homosexuality to investigate their centrality. In the context of attitudinal inference, Study 2 adopted a trait inference paradigm to explore differences in the centrality of components when inferring positive and negative attitudes.

## 2 Study 1: central components of attitudes toward homosexuality in attitudinal measurement

This study aims to investigate the dynamic relationships among components of attitudes toward homosexuality in attitudinal measurement using complex network analysis, thereby identifying central components of attitudes toward homosexuality.

### 2.1 Materials and methods

#### 2.1.1 Participants and procedures

Participants were recruited via Credamo, a widely used and reliable online survey platform in China, and through WeChat, a popular social media App. All participants were Chinese. We excluded individuals who failed attention checks or exhibited abnormal response times (i.e., significantly faster or slower than the average response time), yielding a final sample of 666 valid participants (74.5% women, 75.7% heterosexual). Participants ranged in age from 18 to 40 years, with the modal age group being 18–25 years. No identifiable information, such as name or date of birth, that would allow the principal investigators to readily ascertain the participants’ identity was collected.

#### 2.1.2 Measures

The Two-Factor Attitudes toward Homosexuality Scale (TAHS) was originally developed and administered in Chinese for Study 1. The two factors are prejudice against homosexuality and preference for heterosexuality, each indicated by six items. The TAHS exhibited measurement invariance between heterosexual and non-heterosexual individuals, supporting its use for evaluating attitudes toward homosexuality across the two groups (Guo et al., 2023).

The scale employs a four-point Likert-type response format, with response options ranging from 1 = *strongly agree* to 4 = *strongly disagree*. After reverse-scoring the relevant items, total score ranges from 12 to 48, with each subscale score ranging from 6 to 24. Higher scores indicate more positive attitudes toward homosexuality. The TAHS demonstrated excellent internal consistency among the present sample with a Cronbach’s *α* coefficient of 0.95.

#### 2.1.3 Data analysis

We used SPSS 25.0 (IBM Corp, 2017) to obtain descriptive statistics and conducted complex network analysis using R Version 4.2.2 (R Core Team, 2022).

### 2.2 Results

**Figure 2:**
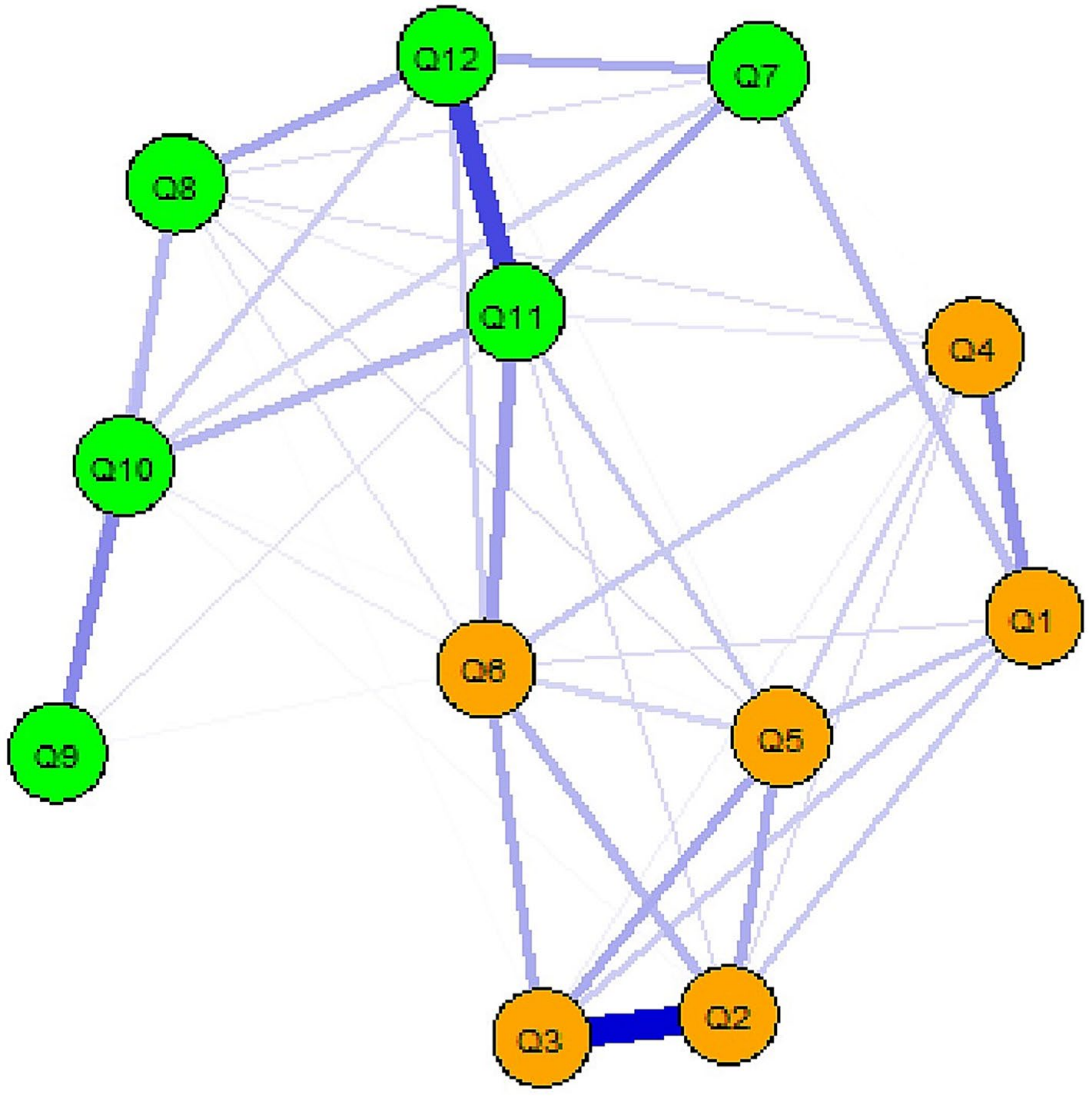
Network of attitudes toward homosexuality.

Mean values and standard deviations of each item in TAHS are shown in Table 1. Network of attitudes toward homosexuality is presented in Figure 2. In this network, nodes Q1–Q6 correspond to items assessing the prejudice against homosexuality factor, and nodes Q7–Q12 correspond to items assessing the preference for heterosexuality factor. Notably, within the prejudice against homosexuality factor, Q5 (“*Homosexuality is an inferior form of sexuality.*”) exhibited the strongest connections with other nodes and occupied a central position. Within the preference for heterosexuality factor, Q11 (“*I try to avoid being friends with homosexual individuals.*”) demonstrated the strongest connections with other nodes and was centrally located within its cluster.

**Table 1:** Mean and standard deviations of each item in the two-factor attitudes toward homosexuality scale.

Item	*Mean*	*SD*
Factor 1: prejudice against homosexuality		
Q1: Homosexual individuals should have equal employment opportunities.	3.61	0.687
Q2: Homosexual individuals should be condemned.	3.64	0.670
Q3: Homosexuality is a sin.	3.69	0.648
Q4: Homosexual individuals should be treated fairly in society.	3.45	0.816
Q5: Homosexuality is an inferior form of sexuality.	3.68	0.613
Q6: Homosexuality is immoral.	3.58	0.690
Factor 2: preference for heterosexuality		
Q7: I can get along with homosexual individuals comfortably.	2.88	0.981
Q8: If homosexual individuals want to be respected, they should remain low-key and avoid being conspicuous.	2.97	0.984
Q9: It is better for homosexual individuals to conceal their sexual orientation.	2.79	0.824
Q10: If homosexual individuals could become heterosexual, they would be happier.	3.08	0.936
Q11: I try to avoid being friends with homosexual individuals.	3.38	0.850
Q12: I cannot accept my relatives being homosexual.	3.19	0.992

SD, Standard Deviation.

A small-world structure was observed in the TAHS network, as indicated by small-world index (*SW*) of 1.03, clustering coefficient (*C*) of 0.70 [0.64, 0.68], and an average length of the shortest paths in the network (*L*) of 1.33 [1.33, 1.33]. To compare the connectivity between factors in the TAHS network, we estimated the shortest path lengths (SPLs) for each factor using the *qgraph* package in R. Dijkstra’s algorithm (Dijkstra, 1959) was employed to determine the shortest path between each pair of nodes, and these distances were averaged to produce a *k* × *k* SPL matrix, with smaller SPL values indicating higher connectivity. Independent samples *t*-test was conducted to compare SPL values for each factor. The results revealed no significant difference in connectivity between the prejudice against homosexuality factor and the preference for heterosexuality factor (*M*_F1_ ± *SD* = 8.37 ± 3.48, *M*_F2_ ± *SD* = 8.08 ± 3.45, *t*(28) = 0.233, *p* = 0.817).

**Figure 3:**
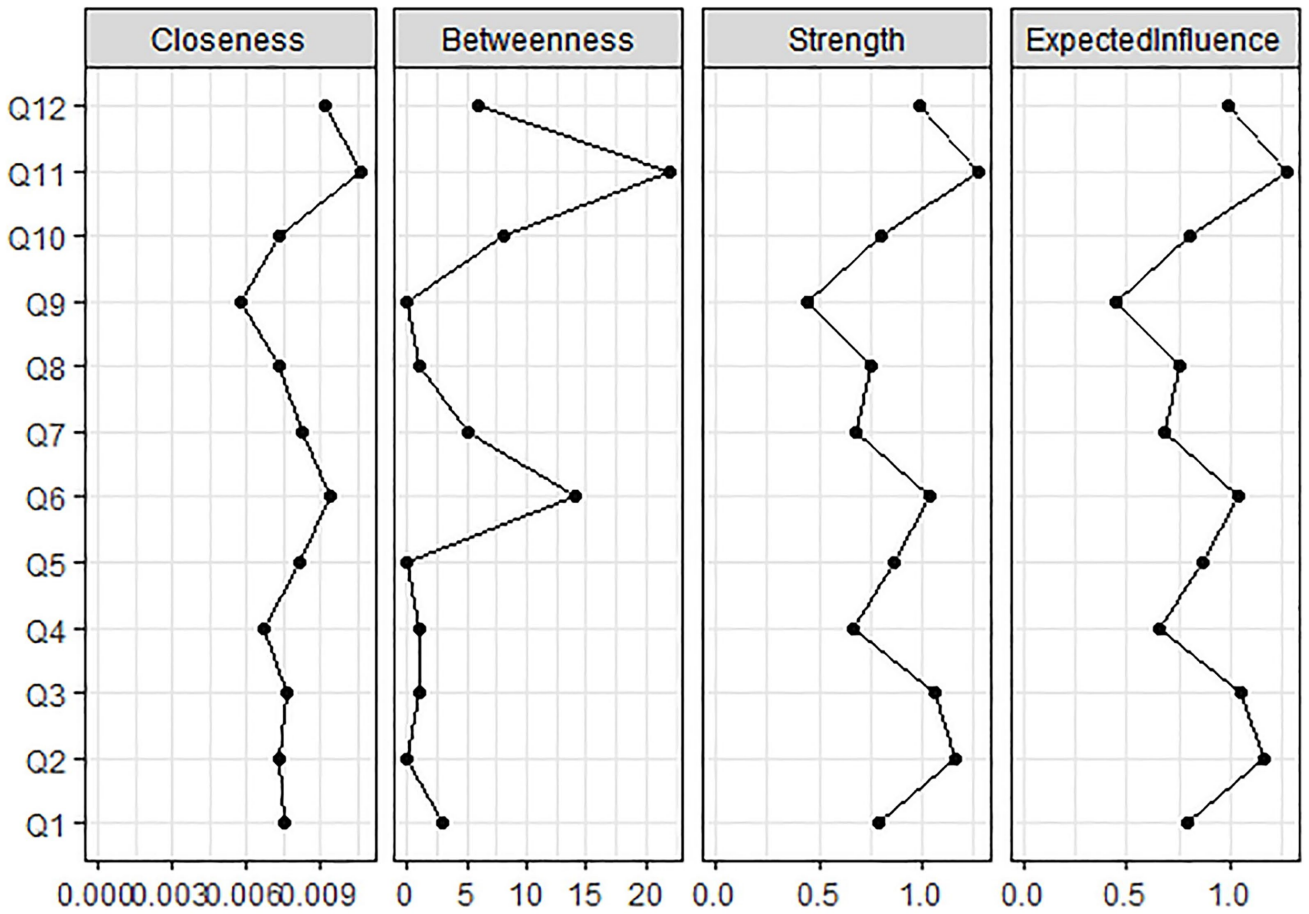
Centrality indices of TAHS network.

Furthermore, node centrality within the TAHS network was evaluated using four indices: closeness, betweenness, strength, and expected influence. As illustrated in Figure 3, item Q11 from the preference for heterosexuality factor (“*I try to avoid being friends with homosexual individuals.*”) consistently exhibited the highest values across all indices, indicating that it is the most central node in the network.

**Figure 4:**
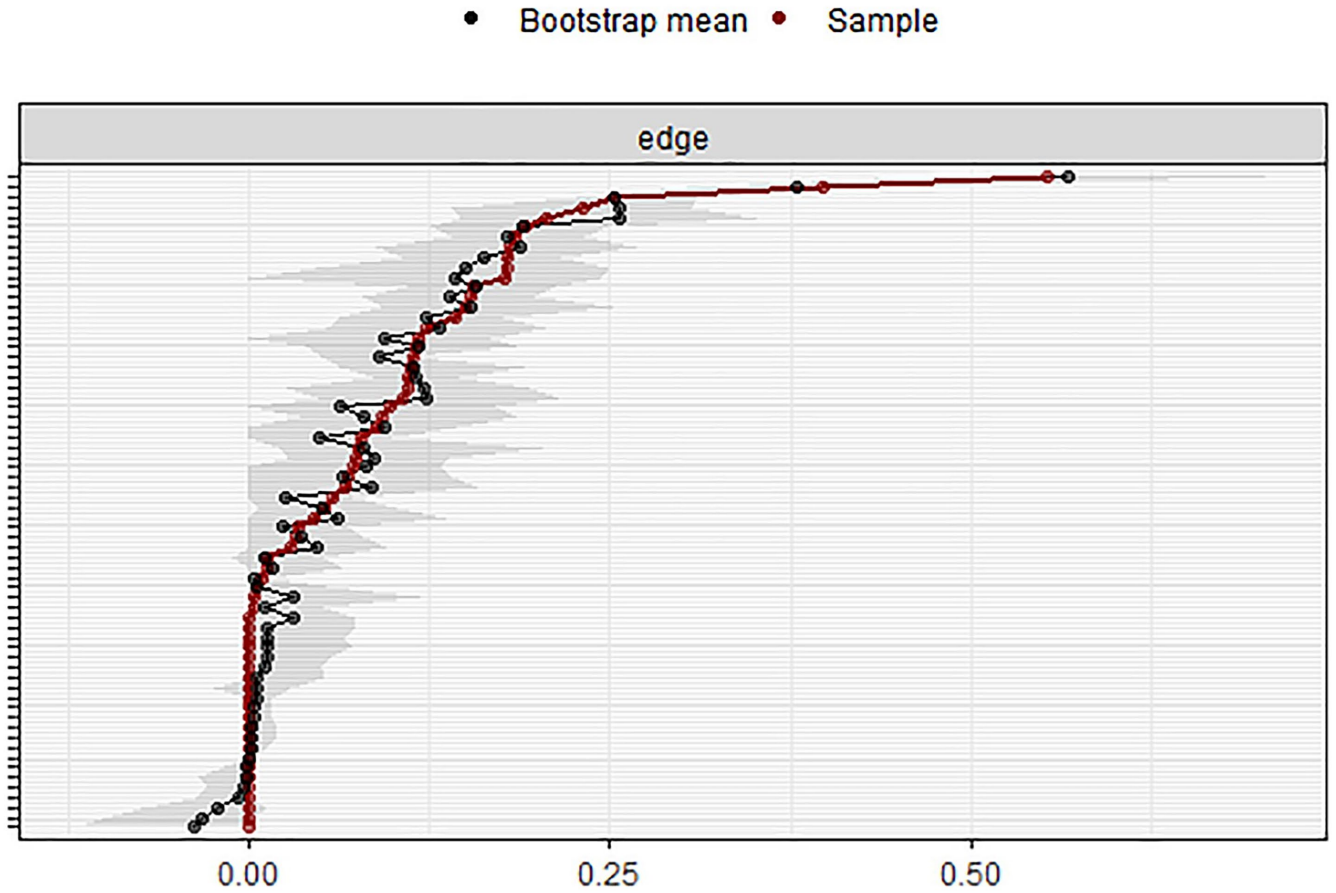
Confidence intervals for TAHS network edge weights.

**Figure 5:**
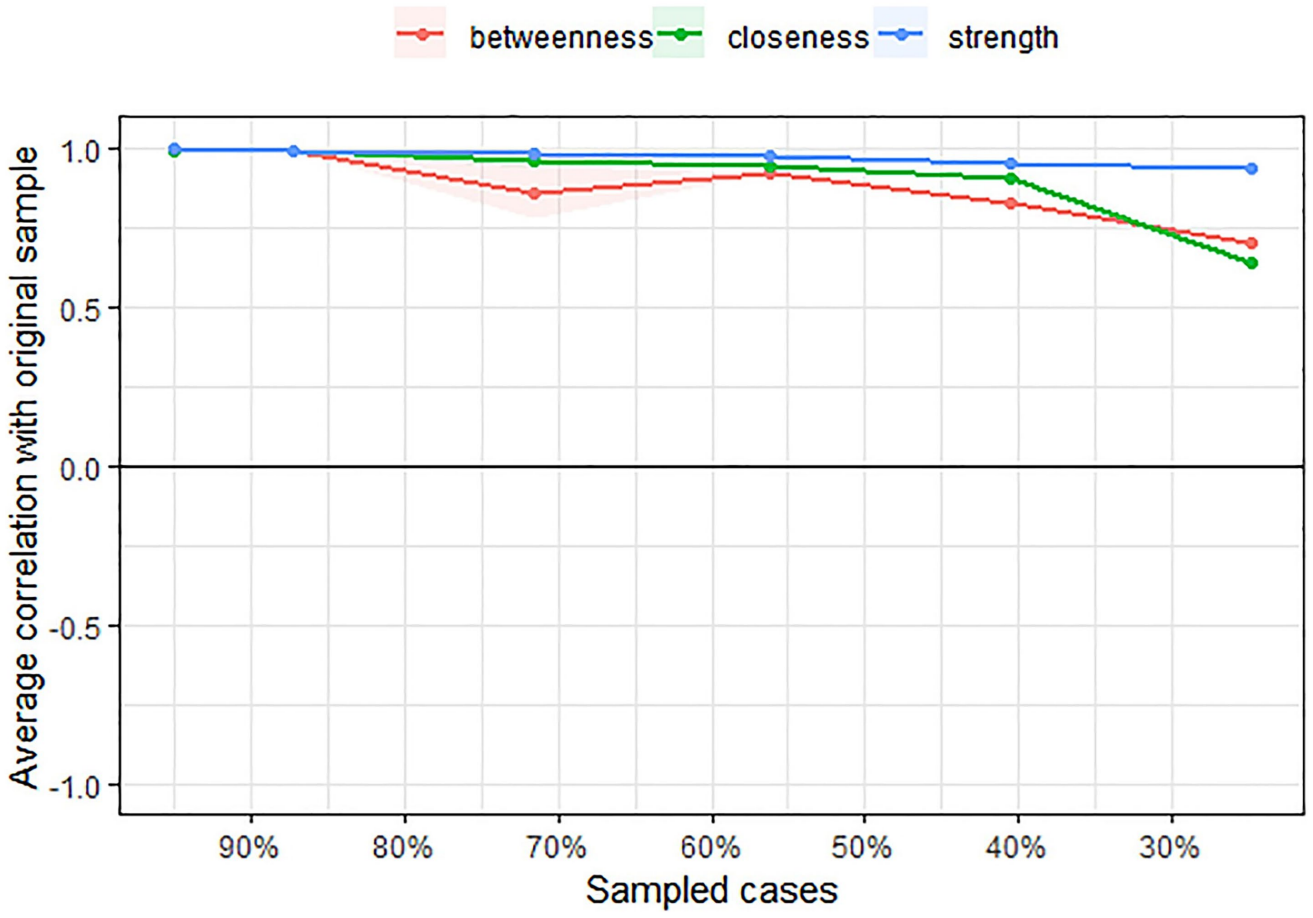
Stability tests of centrality indices of TAHS network.

The stability of the TAHS network was evaluated using the *bootnet* package in R. Figure 4 presents the estimated confidence intervals for the network edge weights, while Figure 5 displays the stability tests for various centrality indices. As illustrated in Figure 5, strength demonstrated relatively high stability across variations in sample size, whereas both closeness and betweenness showed a marked decline in stability when the sample size was reduced.

### 2.3 Discussion

Study 1 applied a complex network analysis approach to construct the network of attitudes toward homosexuality. The results revealed that the TAHS network exhibits a small-world structure, indicating that the two-factor network structure is characterized by high clustering and global connectivity, which facilitates the rapid transmission of information throughout the network. With regard to the difficulty of changing attitudes, no significant difference was found in connectivity between the prejudice against homosexuality factor and the preference for heterosexuality factor. Within the overall TAH network, Q11, representing behavioral tendencies related to social interactions under the preference for heterosexuality factor, was identified as the most central component.

## 3 Study 2: central factors of attitudes toward homosexuality in attitudinal inference

This study aims to investigate the centrality differences in the factors of attitudes toward homosexuality when making inferences about positive or negative attitudes. Using an attitudinal inference research design, we assess centrality based on two key indicators: importance and representativeness.

### 3.1 Study 2a: importance of factors of attitudes toward homosexuality in attitudinal inference

#### 3.1.1 Materials and methods

##### 3.1.1.1 Participants

This study used G*Power 3.1 (Faul et al., 2007) to calculate the required sample size. Given that the primary statistical analysis method employed was paired samples *t*-test, we set the effect size to 0.50, the significance level to 0.05, and the statistical power to 0.95. The estimated sample size was 210.

Participants were recruited through the website of Credamo and WeChat App. All participants were Chinese. After excluding those who failed attention checks or had abnormal response times, the final sample consisted of 259 participants in the negative inference group (59.1% women; 70.3% heterosexual; inclusion rate was 94.2%) and 269 participants in the positive inference group (57.1% women; 67.3% heterosexual; inclusion rate was 97.8%). Participants’ ages ranged from 18 to 56 years, with the modal age group being 18–28 years.

##### 3.1.1.2 Measures and procedures

**Figure 6:**
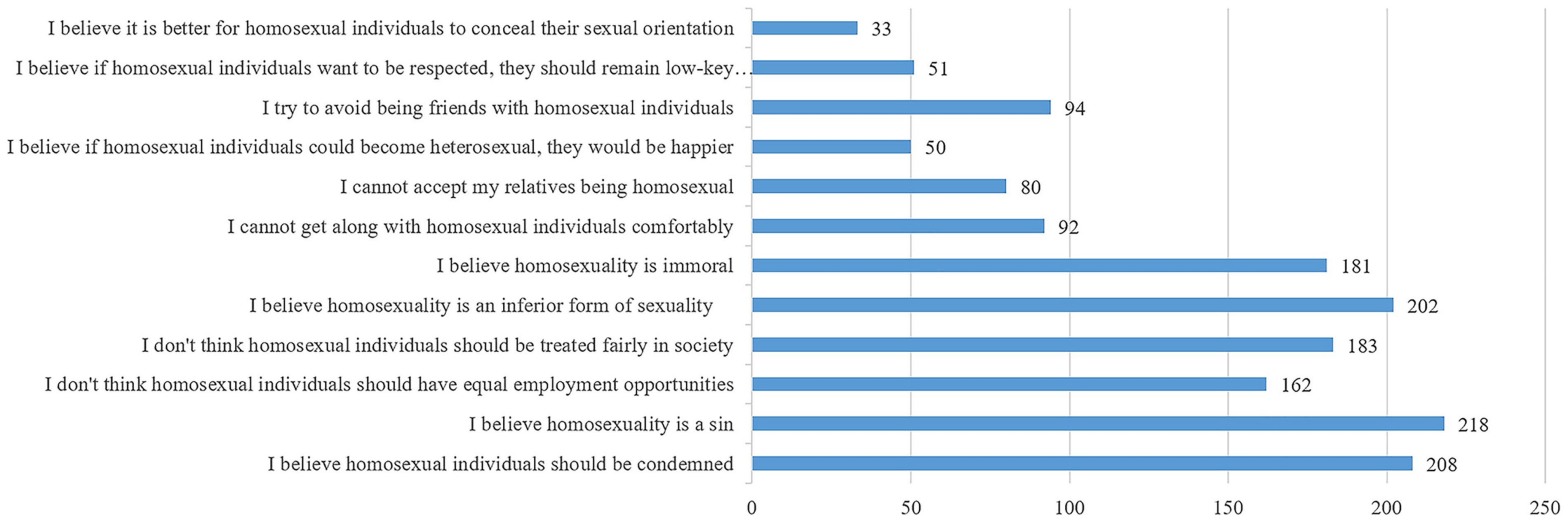
Item selection frequency for inferring negative attitudes toward homosexuality.

**Figure 7:**
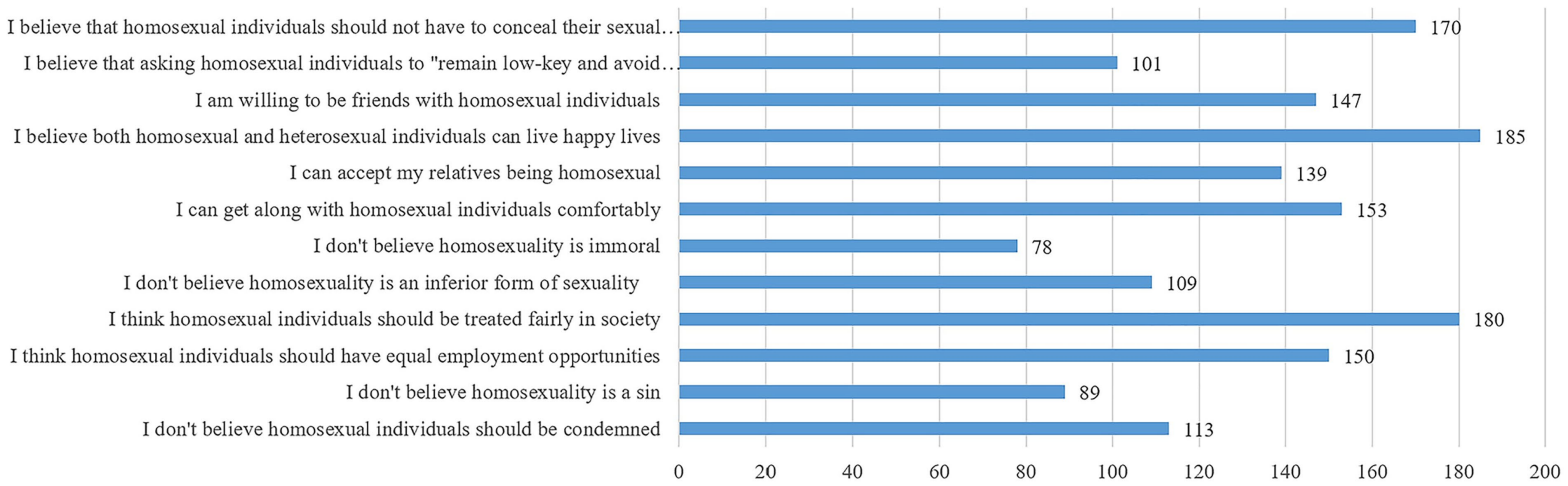
Item selection frequency for inferring positive attitudes toward homosexuality.

The measurement instrument included six basic demographic questions, a questionnaire adapted from the 12-item TAHS to assess the importance of different factors in inferring positive or negative attitudes (see Figures 6, 7), and two attention check questions. To minimize potential order effects, the 12 statements representing positive or negative attitudes were presented in a randomized sequence. The questionnaires were administered in Chinese.

Taking the importance questionnaire for inferring positive attitudes as an example, participants were provided with the following instructions:

*“Hello, this survey aims to assess how you* ***infer others’ attitudes toward homosexuality*** *; it is not an assessment of your own attitudes toward homosexuality. In your opinion, when a person exhibits the following behaviors or viewpoints, which statements best help you determine that they hold* ***a positive attitude toward homosexuality*** *? Please select six statements from the 12 listed below that you believe indicate the most positive attitudes toward homosexuality.* ***You must select exactly six—no more, no fewer.*** *”*

Each statement appeared in the following format: *“Respondent: I can get along with homosexual individuals comfortably.”* This phrasing was intentionally chosen to encourage participants to interpret the item as a statement made by another person (i.e., the “respondent”) and to prompt them to make an objective inference about that person’s attitudes, rather than to express their own. To encourage participants properly understood the inferential nature of the task, we implemented a manipulation check item instructing them to: “Please confirm you recognize this survey assesses your inferences about others’ attitudes rather than your personal attitudes by selecting (−3).” All task materials from Study 2 that were shown to participants are included in the Supplementary materials.

To assess the perceived importance of different items (beliefs, affective responses, and behavioral tendencies) when participants infer others’ attitudes, we implemented a forced-choice paradigm. This approach required participants to weigh the relative importance of various belief, affective, and behavioral components of others’ attitudes, with selection frequency serving as the indicator to differentiate between more and less important items (Cottrell et al., 2007; Jiao et al., 2022). Positive attributes are more difficult to confirm than negative ones (Klein and O’Brien, 2016), and guided by Guo et al.’s (2023) two-factor three-class model, we predicted that the preference for heterosexuality factor—due to its benevolent bias—might introduce greater ambiguity in attitude inference compared to the more overt prejudice against homosexuality factor. We therefore hypothesized that the perceived importance of prejudice against homosexuality factor and preference for heterosexuality factor would differ when making attitude inferences.

Since each factor comprises six items, Study 2a required participants to select the six most diagnostic items (out of twelve) for attitude inference. The selection frequency of statements under the prejudice against homosexuality factor and the preference for heterosexuality factor was calculated separately.

In Study 2a, we employed the attitude factors (prejudice against homosexuality vs. preference for heterosexuality) as independent variables, with the mean selection frequency of each factor serving as the dependent variable. The study further analyzed differences in how participants of varying sex assigned at birth (male/female) and sexual orientations (heterosexual/non-heterosexual) evaluated the importance of these indicators during attitude inference. We included the variable of sex assigned at birth because prior research indicates that attitudes toward homosexuality often vary significantly based on an individual’s sex assigned at birth (e.g., males tend to report more negative attitudes toward homosexuality than females; Guo and Liu, 2019). Sexual orientation was also included as a core variable to enable comparisons between attitudes of the out-group (heterosexual individuals) and the in-group (sexual minority individuals).

##### 3.1.1.3 Data analysis

The study examined positive and negative attitude inferences using importance ratings as the dependent variable. A mixed-design approach was employed, with factor categories as within-subjects variables and participants’ sex assigned at birth and sexual orientation as between-subjects variables to facilitate subsequent data analysis. A paired samples *t*-test was conducted to compare the importance of factors. To further examine differences in sex assigned at birth and sexual orientation in the inference of negative and positive attitudes, a two-way ANOVA was performed. All data analyses were conducted using SPSS 25.0 (IBM Corp, 2017).

#### 3.1.2 Results

For the inference of negative attitudes toward homosexuality, Figure 6 presents the selection frequency of each statement used to infer negative attitudes. Results from the paired samples *t*-test indicated that the mean selection frequency of statements under the prejudice against homosexuality factor was significantly higher than that under the preference for heterosexuality factor (*M*_F1_ ± *SD* = 4.46 ± 1.49, *M*_F2_ ± *SD* = 1.54 ± 1.90; *t*(258) = 15.70, *p* < 0.001, Cohen’s *d* = 1.96). This finding suggests that when making negative inferences, the prejudice against homosexuality factor is more important than the preference for heterosexuality factor.

A two-way ANOVA was conducted to further examine differences in the importance of the prejudice against homosexuality factor across sex assigned at birth and sexual orientation groups. Results showed that the main effect of sex assigned at birth was marginally significant but had a small effect size, suggesting that the sex difference may not be practically meaningful (*M*_M_ₐₗₑ ± *SD* = 4.25 ± 1.45, *M*_Female_ ± *SD* = 4.59 ± 1.51; *F*(1, 255) = 3.23, *p* = 0.074, *η*^2^ₚ = 0.012). The main effect of sexual orientation was not significant (*F*(1, 255) = 0.03, *p* = 0.864), nor was the interaction effect between sex assigned at birth and sexual orientation (*F*(1, 255) = 0.17, *p* = 0.682).

For the inference of positive attitudes toward homosexuality, Figure 7 presents the selection frequency of each statement used to infer positive attitudes. Results from the paired samples *t*-test indicated that the mean selection frequency of statements under the preference for heterosexuality factor was significantly higher than that under the prejudice against homosexuality factor (*M*_F1_ ± *SD* = 2.67 ± 1.30, *M*_F2_ ± *SD* = 3.33 ± 1.30; *t*(268) = −4.13, *p* < 0.001, Cohen’s *d* = 0.51). This finding suggests that when making inferences of positive attitudes, the preference for heterosexuality factor is more important than the prejudice against homosexuality factor.

In terms of differences across sex assigned at birth and sexual orientation groups in positive inferences, two-way ANOVA showed that the main effect of sex assigned at birth in the mean selection frequency of statements under the preference for heterosexuality factor was marginally significant but had a small effect size, suggesting that the sex difference may not be practically meaningful (*M*_M_ₐₗₑ ± *SD* = 3.54 ± 1.30, *M*_Female_ ± *SD* = 3.17 ± 1.29; *F*(1, 265) = 3.77, *p* = 0.053, *η*^2^ₚ = 0.014). The main effect of sexual orientation was significant (*F*(1, 265) = 13.08, *p* < 0.001), such that non-heterosexual participants rated the importance of preference for heterosexuality factor as significantly higher compared to heterosexual participants (*M*_Het_ ± *SD* = 3.12 ± 1.33, *M*_Non-het_ ± *SD* = 3.75 ± 1.13). The interaction effect between sex assigned at birth and sexual orientation was not significant (*F*(1, 265) = 0.03, *p* = 0.853).

### 3.2 Study 2b: representativeness of factors of attitudes toward homosexuality in attitudinal inference

Study 2b explored the centrality of factors of attitudes toward homosexuality in attitudinal inference using representativeness as an indicator. In Study 2a in which importance was used as an indicator of centrality, the effect size of sex difference was small. Therefore, in Study 2b, only participants’ sexual orientation was considered when examining differences in factor representativeness.

#### 3.2.1 Materials and methods

##### 3.2.1.1 Participants

This study used G*Power 3.1 (Faul et al., 2007) to calculate the required sample size. Given that the primary statistical analysis method employed was two-way ANOVA, we set the effect size to 0.25, the significance level to 0.05, and the statistical power to 0.95. The estimated sample size was 158.

Participants were recruited through the website of Credamo and WeChat App. All participants were Chinese. After excluding those who failed attention checks or had abnormal response times, the final sample consisted of 231 participants in the negative inference group (61.5% women; 68.0% heterosexual; inclusion rate was 92.4%) and 161 participants in the positive inference group (55.9% women; 48.4% heterosexual; inclusion rate was 80.5%). Participants’ ages ranged from 18 to 56 years, with the modal age group being 18–28 years.

##### 3.2.1.2 Measures and procedures

The measurement instrument included six basic demographic questions, a questionnaire adapted from the 12-item TAHS to assess the representativeness of different factors in inferring positive or negative attitudes, and two attention check questions. To minimize potential order effects, the 12 statements representing positive or negative attitudes were presented in a randomized sequence. The questionnaires were administered in Chinese.

The adapted TAHS-Negative Attitude Inference Questionnaire demonstrated excellent internal consistency, with a Cronbach’s *α* coefficient of 0.891. A confirmatory factor analysis (CFA) was conducted on the Negative Attitude Inference Questionnaire to examine its two-factor structure. The model fit indices were as follows: *χ*^2^ = 145.49, *df* = 53, χ^2^/*df* = 2.75, RMSEA (90% CI) = 0.074 [0.068, 0.084], CFI = 0.899, TLI = 0.861, and SRMR = 0.078. Although the CFI and TLI fell slightly below the conventional cutoff of 0.90, the ratio of chi-square to degrees of freedom (χ^2^/df) remained below 3.0, and the RMSEA fell within the acceptable range for reasonable model fit. Additionally, the SRMR approached the commonly referenced upper bound of 0.08, collectively supporting adequate model acceptability. This study prioritizes theoretical coherence and parameter interpretability, and although the fit indices were not optimal, they nevertheless lie within an acceptable range. Therefore, we concluded that the model is appropriate for subsequent analyses. Standardized factor loadings ranged from 0.710 to 0.880 for Factor 1 and from 0.658 to 0.829 for Factor 2.

Based on the factor analysis results, one item from the preference for heterosexuality factor was removed from the adapted TAHS-Positive Attitude Inference Questionnaire. The revised Positive Attitude Inference Questionnaire comprised 11 items, with a Cronbach’s α coefficient of 0.787. A CFA was conducted on the Positive Attitude Inference Questionnaire to examine its two-factor structure. The model fit indices were as follows: *χ*^2^ = 101.84, *df* = 43, *χ*^2^/*df* = 2.37, RMSEA (90% CI) = 0.092 [0.069, 0.115], CFI = 0.883, TLI = 0.851, and SRMR = 0.086. Although the fit indices were not optimal, they nevertheless lie within an acceptable range. Therefore, we concluded that the model is appropriate for subsequent analyses. Standardized factor loadings ranged from 0.598 to 0.814 for Factor 1 and from 0.505 to 0.760 for Factor 2.

Reliability analyses and CFA confirmed that the Negative and Positive Attitude Inference Questionnaires in Study 2b had adequate reliability and construct validity, making them appropriate for measuring attitude inferences. To ensure the content validity of the study, we implemented two procedures. First, we invited four psychology experts—with backgrounds in statistics, psychological counseling, and sexuality psychology—to evaluate the content of the Negative and Positive Attitude Inference Questionnaires. Second, we implemented a manipulation check item to ensure participants properly understood the inferential nature of the task, which instructed them to: “Please confirm you recognize this survey assesses your inferences about others’ attitudes rather than your personal attitudes by selecting (−3).”

Taking the representativeness questionnaire for inferring positive attitudes as an example, participants were provided with the following instructions:

*“Hello, this survey aims to assess how you* ***infer others’ attitudes toward homosexuality*** *; it is not an assessment of your own attitudes toward homosexuality. Based on your personal experiences and perspectives, please evaluate the extent to which each of the following behaviors or viewpoints represents a positive or negative attitude toward homosexuality. You will rate each item* ***on a scale from −5 to +5*** *, where positive scores indicate a positive attitude, negative scores indicate a negative attitude, and a score of 0 means it is difficult to determine. The larger the absolute value, the stronger the perceived positivity or negativity.”*

To assess the representativeness of different items (beliefs, affective responses, and behavioral tendencies) when participants infer others’ attitudes, participants were required to rate each item (Goodwin et al., 2014; Jiao et al., 2022). Given that certain manifestations of the preference for heterosexuality factor may carry subjective benevolence (e.g., “If homosexual individuals could become heterosexual, they would achieve greater happiness”; Guo et al., 2023), some participants might interpret such beliefs as representing positive attitudes based on their subjectively kind intentions, while others might judge them as reflecting negative attitudes due to their detrimental effects on sexual minorities. Therefore, the representativeness rating scale was designed to range from −5 to +5.

In Study 2b, we employed the attitude factors (prejudice against homosexuality vs. preference for heterosexuality) as independent variables, with the mean representativeness ratings of manifestations of these factors serving as the dependent variable. The study further examined how this representativeness evaluations differed across participants of varying sexual orientations (heterosexual vs. non-heterosexual) during attitude inference.

##### 3.2.1.3 Data analysis

The study examined positive and negative attitude inferences using representativeness ratings as the dependent variable. A mixed-design approach was employed, with factor categories as within-subjects variables and participants’ sexual orientation as between-subjects variable to facilitate subsequent data analysis. We calculated the item-level means for both positive and negative representativeness ratings. A two-way ANOVA was conducted to examine the effects of factor category (prejudice against homosexuality vs. preference for heterosexuality, within-subjects) and sexual orientation (heterosexual vs. non-heterosexual, between-subjects) on the representativeness ratings. All data analyses were conducted using SPSS 25.0 (IBM Corp, 2017).

#### 3.2.2 Results

For the inference of negative attitudes toward homosexuality, descriptive statistics for the representativeness ratings of the prejudice against homosexuality and preference for heterosexuality factors are presented in Tables 2, 3. Results from the two-way ANOVA revealed a significant main effect of factor category, with prejudice against homosexuality factor being rated as significantly more representative of negative attitudes than preference for heterosexuality factor (*F*(1, 229) = 545.6, *p* < 0.001, *η*^2^ = 0.70). There was also a significant main effect of sexual orientation (*F*(1, 229) = 67.18, *p* < 0.001, *η*^2^ = 0.25), as well as a significant interaction effect between factor category and sexual orientation (*F*(1, 229) = 37.78, *p* < 0.001, *η*^2^ = 0.05).

**Table 2:** Descriptive statistics for the representativeness ratings of negative representativeness ratings.

Factor	Item	*M*	*SD*
Prejudice against homosexuality	I believe homosexual individuals should be condemned	−3.69	1.70
	An individual believes homosexuality is a sin	−3.71	1.75
	I do not think homosexual individuals should have equal employment opportunities	−3.95	1.44
	I do not think homosexual individuals should be treated fairly in society	−3.86	1.56
	I believe homosexuality is an inferior form of sexuality	−4.06	1.38
	I believe homosexuality is immoral	−3.71	1.75
Preference for heterosexuality	I cannot get along with homosexual individuals comfortably	−1.71	2.42
	I cannot accept my relatives being homosexual	−1.49	2.71
	I believe if homosexual individuals could become heterosexual, they would be happier	−0.94	2.93
	I try to avoid being friends with homosexual individuals	−2.45	2.14
	I believe if homosexual individuals want to be respected, they should remain low-key and avoid being conspicuous	−0.13	2.87
	I believe it is better for homosexual individuals to conceal their sexual orientation to conceal their sexual orientation	−0.22	2.68

**Table 3:** Descriptive statistics for the representativeness ratings of different factors when inferring negative attitudes toward homosexuality.

Factor	Sexual orientation	*n*	*M*	*SD*
Prejudice against homosexuality	Heterosexual	157	−3.62	1.17
	Non-heterosexual	74	−4.45	0.77
Preference for heterosexuality	Heterosexual	157	−0.47	1.94
	Non-heterosexual	74	−2.61	1.57

Group differences were further analyzed with Sidak-adjusted *post hoc* tests (*α* = 0.05). Regarding sexual orientation differences, non-heterosexual participants rated statements under the prejudice against homosexuality factor as significantly more representative of negative attitudes than heterosexual participants (*t*(458) = 3.95, *p* < 0.001, Cohen’s *d* = 0.78). Non-heterosexual participants also rated statements under the preference for heterosexuality factor as significantly more representative of negative attitudes than heterosexual participants (*t*(458) = 10.18, *p* < 0.001, Cohen’s *d* = 1.17). For differences in factor category, heterosexual participants rated statements under the prejudice against homosexuality factor as significantly more representative of negative attitudes than those under the preference for heterosexuality factor (*t*(229) = 26.06, *p* < 0.001, Cohen’s *d* = 1.97). Likewise, non-heterosexual participants also rated statements under the prejudice against homosexuality factor as significantly more representative of negative attitudes than those under the preference for heterosexuality factor (*t*(229) = 10.44, *p* < 0.001, Cohen’s *d* = 1.49).

For the inference of positive attitudes toward homosexuality, descriptive statistics for the representativeness ratings of the prejudice against homosexuality and preference for heterosexuality factors are presented in Tables 4, 5. Results from the two-way ANOVA revealed a significant main effect of factor category, with the preference for heterosexuality factor being rated as significantly more representative of positive attitudes than the prejudice against homosexuality factor (*F*(1, 159) = 7.40, *p* = 0.007, *η*^2^ = 0.67). There was no significant main effect of sexual orientation (*F*(1, 159) = 1.42, *p* = 0.235). The interaction effect between factor category and sexual orientation was not significant (*F*(1, 159) = 0.79, *p* = 0.376).

**Table 4:** Descriptive statistics for the representativeness ratings of positive representativeness ratings.

Factor	Item	*M*	*SD*
Prejudice against homosexuality	I do not believe homosexual individuals should be condemned	3.46	1.43
	I do not believe homosexuality is a sin	3.12	1.79
	I think homosexual individuals should have equal employment opportunities	3.71	1.32
	I think homosexual individuals should be treated fairly in society	3.43	1.33
	I do not believe homosexuality is an inferior form of sexuality	3.53	1.50
	I do not believe homosexuality is immoral	3.21	1.57
Preference for heterosexuality	I can get along with homosexual individuals comfortably	3.43	1.33
	I can accept my relatives being homosexual	3.81	1.13
	I believe both homosexual and heterosexual individuals can live happy lives	4.03	1.23
	I am willing to be friends with homosexual individuals	3.81	1.13
	I believe that homosexual individuals should not have to conceal their sexual orientation, living authentically is better for them	3.37	1.67

**Table 5:** Descriptive statistics for the representativeness ratings of different factors when inferring positive attitudes toward homosexuality.

Factor	Sexual orientation	*n*	*M*	*SD*
Prejudice against homosexuality	Heterosexual	78	3.47	0.99
	Non-heterosexual	83	3.54	1.24
Preference for heterosexuality	Heterosexual	78	3.63	0.65
	Non-heterosexual	83	3.86	0.78

Group differences were further analyzed with Sidak-adjusted *post hoc* tests (α = 0.05). The results indicated that, regarding differences in sexual orientation, there was no significant difference between heterosexual and non-heterosexual participants in their positive representativeness ratings of statements under the prejudice against homosexuality factor (*t*(318) = 0.45, *p* = 0.878) or the preference for heterosexuality factor (*t*(318) = 1.49, *p* = 0.257). Regarding differences in factor categories, heterosexual participants did not show a significant difference between their positive representativeness ratings of statements under the prejudice against homosexuality factor and those under the preference for heterosexuality factor (*t*(159) = 1.28, *p* = 0.366). However, non-heterosexual participants rated statements under the preference for heterosexuality factor as significantly more representative of positive attitudes than those under the prejudice against homosexuality factor (*t*(159) = 2.59, *p* = 0.021, Cohen’s *d* = 0.31).

### 3.3 Discussion

Based on the two-factor attitudes toward homosexuality model, the current study explored the central factors for inferring negative and positive attitudes. Using importance and representativeness as key indicators, the results revealed that when inferring negative attitudes, prejudice against homosexuality factor was more important and representative than preference for heterosexuality factor. Conversely, when inferring positive attitudes, the preference for heterosexuality factor was more important and representative than the prejudice against homosexuality factor. These findings suggest that the centrality of factors within attitudes toward homosexuality varies by valence. Additionally, different sexual orientation groups may evaluate the centrality of the same factor differently when making attitudinal inferences.

## 4 General discussion

In the culture of human sexuality, sexual minority and heterosexual individuals constantly engage in intergroup interactions within a dynamic landscape shaped by mainstream and subcultural norms. The two-factor structure of attitudes toward homosexuality—comprising both *prejudice against homosexuality* factor and *preference for heterosexuality* factor—holds substantial importance for comprehending the socio-cultural construction of such attitudes.

Based on the two-factor attitudes toward homosexuality model, this study examined component-based and factor-based centrality in attitudes towards homosexuality through two studies. Study 1 employed complex network analysis to investigate the dynamics among components of self-reported attitudes toward homosexuality, revealing that the component pertaining to social interaction under the *preference for heterosexuality* factor occupied the most central position in the attitude network. Study 2 consisted of two sub-studies examining how attitudinal inferences toward homosexuality varied by valence. The results showed that when making inferences about negative attitudes toward homosexuality, the factor *prejudice against homosexuality* was more salient and representative. In contrast, when inferring positive attitudes toward homosexuality, preferences for heterosexuality factor demonstrated greater importance and representativeness.

### 4.1 Central components in attitudes toward homosexuality

The components of attitudes toward homosexuality do not exist in a simple, parallel relationship, but rather exhibit hierarchical distinctions in centrality and marginality. The most central component (i.e., the item exerting the highest influence on the attitude network) was found to be social interaction tendencies under *preference for heterosexuality* factor. This specific component demonstrates a leveraging effect, wherein change in one component can induce broader, holistic shifts in the overall attitude. These findings may help explain, to some extent, the effectiveness of intergroup contact-based interventions in improving attitudes toward homosexuality (Heinze and Horn, 2009; Sakalh and Ugurlu, 2002).

Intergroup Contact Theory posits that intergroup prejudice stems from either insufficient information or misinformation about an outgroup. Intergroup contact provides opportunities to acquire new information and correct erroneous beliefs (Allport, 1954). Within the network of attitudes toward homosexuality, manifestations pertaining to social interaction occupy a more central and influential position. This structural characteristic suggests that targeted interventions focusing on these pivotal nodes could potentially yield maximal outcomes with minimal input. For instance, Guo et al. (2021) designed two experimental conditions: one where participants were informed about forthcoming contact with gay men or lesbians prior to interaction (pre-contact disclosure group), and another where this information was disclosed after contact (post-contact disclosure group). The results demonstrated that participants exhibited greater improvements in attitudes toward homosexuality in the post-contact disclosure condition compared to the pre-contact disclosure condition. One possible explanation is that participants hold negative attitudes toward homosexuality may have heightened vigilance and defensive responses when informed in advance about contact with sexual minority people, thereby compromising the effectiveness of the intergroup contact intervention. In contrast, participants in the post-contact disclosure group may experience attitudinal shifts through engagement with the most central attitudinal component, social interaction tendencies, leading to more comprehensive attitude improvements.

The study 1 investigated the central components of attitudes toward homosexuality, aiming to enrich theoretical understanding of the attitude network structure. However, it is worth noting that focusing solely on these central components would be insufficient when examining attitudes toward homosexuality, as within the complete attitudinal network, changes in any single node could potentially exert influence on other interconnected components.

### 4.2 Inference of attitude toward homosexuality: positive or negative?

Meyer’s (2003) Minority Stress Model provides a theoretical framework for understanding the mental health disparities experienced by sexual minority populations. This theoretical framework postulates three distinct stress processes: (1) objective/external stressors, encompassing institutional discrimination along with prejudice and violence in direct interpersonal interactions; (2) expected stigma, where individuals anticipate potential victimization and maintain vigilance; and (3) internalization of societal negative attitudes. Notably, both the first and second stress processes involve sexual minority individuals’ inferences about others’ attitudes toward homosexuality within heteronormative cultural contexts. Parallel inference processes similarly occur when heterosexual individuals assess others’ attitudes toward homosexuality in daily life. Existing research has demonstrated that heterosexual individuals exhibit greater willingness to form friendships with sexual minority people when they perceive their environment as LGBTQ+-friendly (Paul Poteat et al., 2009). However, empirical studies have demonstrated that the fragility of heterosexual identity—a construct contingent upon the systematic exclusion and symbolic denial of non-heterosexual identities to preserve its dominant status (Bosson and Vandello, 2011) —may drive heightened social distancing behaviors toward sexual minorities when individuals perceive threats to their heterosexual identity (Talley and Bettencourt, 2008). This reaction may reflect concerns that expressing positive attitudes toward homosexuality could lead others to infer that the individual is a sexual minority individual.

To investigate the centrality of different factors when inferring others’ attitudes toward homosexuality, Study 2 employed two indicators (i.e., importance and representativeness) to examine this phenomenon. The results revealed valence-dependent centrality patterns: when inferring negative attitudes, *prejudice against homosexuality* emerged as the more central factor; whereas for positive attitude inferences, *preference for heterosexuality* demonstrated greater centrality.

The inference of attitudes toward homosexuality exhibited sexual orientation differences. Specifically, non-heterosexual participants perceived both *prejudice against homosexuality* and *preference for heterosexuality* as significantly more negative than did heterosexual participants. This pattern may reflect an ingroup-outgroup sensitivity bias: as non-heterosexual individuals (as attitude holders) maintain closer social proximity to the attitude target (homosexuality) compared to heterosexual individuals, they demonstrate heightened sensitivity to negative manifestations. Conversely, heterosexual individuals, as outgroup members, may systematically underestimate the harmful implications of such attitudes (a group-serving bias effect; Pettigrew, 1979, 1980). Notably, non-heterosexual participants showed greater awareness of the negativity embedded in external expressions associated with *preference for heterosexuality*, which may stem from the “subjective benevolence” often embedded in the expressions of heterosexual preference (Hoffarth and Hodson, 2014; e.g., “LGBT individuals should maintain low profiles to gain respect”). Such ostensibly well-intentioned expressions may inadvertently obscure the underlying social inequality between attitude holders and targets.

Moreover, results of positive attitude inferences revealed divergent evaluation patterns: heterosexual participants showed no significant difference in their assessments of the positive representativeness between low-level *prejudice against homosexuality* and low-level *preference for heterosexuality* expressions. In contrast, non-heterosexual participants rated low-level *preference for heterosexuality* expressions as significantly more positively representative than comparable *prejudice against homosexuality* expressions. These findings align with Matsick et al. (2025), who emphasize that sexual minority individuals often experience seemingly positive attitudes that, in practice, reinforce bias during interactions with heterosexual individuals who uphold dominant-group privilege. Thus, sexual minority people may become particularly attuned to distinguishing messages of genuine, unconditional support from those that merely lack overt hostility.

### 4.3 Implications

While prior research on attitudes toward homosexuality has predominantly focused on attitudinal structures, the current study advances the field by identifying central components and factors within these structures, offering a novel theoretical framework. Theoretically, this contribution enables researchers to develop a more systematic and multidimensional understanding of attitudes toward homosexuality.

Practically, it yields two key implications: (1) For improving attitudes toward homosexuality, identifying central components in attitudinal network allows practitioners to target pivotal components for more efficient and effective interventions; (2) Understanding the centrality differences in factors underlying attitudinal inferences toward homosexuality can help individuals recognize the negative manifestations embedded in high-level *preference for heterosexuality* expressions and differentiate between the relative positivity of low-level *prejudice against homosexuality* and *preference for heterosexuality* manifestations. This, in turn, raises awareness of the detrimental effects of ostensibly benevolent yet ultimately prejudicial attitudes (Guo et al., 2023; Matsick et al., 2025). These distinctions provide opportunities to examine unique contributions of specific attitudinal factors in predicting unique health outcomes among sexual minority people. Moreover, they highlight nuanced intervention targets for reducing homonegativity and carry significant implications for advancing social equity for sexual minority populations.

### 4.4 Limitations

This study investigated the centrality of attitudes toward homosexuality through both attitude measurement and inference paradigms. However, several limitations should be acknowledged.

First, while the current research examined centrality differences among components and factors within a two-factor model of attitudes toward homosexuality, future studies could explore how individual differences and sociocultural factors interact with the central components and factors of these attitudes.

Second, the reliance on self-report questionnaires, despite incorporating lie-detection items and minimum response time thresholds to enhance data reliability, may still introduce discrepancies between reported and actual attitudes.

Third, in Study 2, the phrasing of the attitudinal inference statements may have inadvertently activated participants’ self-referential processes, potentially compromising the construct validity of the task, which was intended to measure inferences about others’ attitudes. Although each attitudinal inference item was explicitly framed as a self-report from another person to reduce such bias, future research would benefit from implementing pilot studies or incorporating a manipulation check to ensure that participants clearly understand the task as one involving attitudinal inference rather than personal evaluation.

Finally, the online sampling approach used in this study recruited a disproportionately heterosexual sample, which may have led to uneven representation across demographic subgroups. Consequently, the generalizability of our findings requires verification in more diverse populations.

## 5 Conclusion

The components and factors within attitudes toward homosexuality exhibit distinct patterns of centrality, characterized by the following specific features:

1. Structural Centrality: Within the complex network model, components representing social interaction tendencies under the *preference for heterosexuality* factor emerge as pivotal hubs, occupying the most central positions in the attitudinal network.
2. Valence-Dependent Centrality in Attitude Inference: For *negative attitude inferences,* high levels of *prejudice against homosexuality* demonstrate greater importance and representativeness than high levels of *preference for heterosexuality*. For positive attitude inferences, low levels of *preference for heterosexuality* are perceived as more important and representative than low levels of *prejudice against homosexuality*.
3. Sexual Orientation Differences in Inference: Compared to heterosexual individuals, non-heterosexual individuals evaluate both *prejudice against homosexuality* and *preference for heterosexuality* as significantly more negative, reflecting heightened sensitivity to attitudinal bias.

Based on these findings, our study provides a network-based and inferential perspective on the structure of attitudes toward homosexuality. This approach not only deepens the theoretical understanding of attitudinal centrality but also offers practical insights for interventions aimed at reducing bias and improving intergroup relations.

## Data Availability

The raw data supporting the conclusions of this article will be made available by the authors, without undue reservation.

## Footnotes

^1^The index *SW* is used to evaluate whether a network has high clustering and high global connectivity, meaning all nodes are closely connected, and when one node changes, other nodes also change. The index *L* is an indicator that evaluates the average length of paths between nodes. The shorter the path length between nodes, the closer the connections. The index *C* is calculated as the number of closed triangular node group patterns formed in the network (i.e., three mutually closely connected nodes) divided by the number of connected triplets (where these connections are not necessarily all mutually related).

## Author’s note

We acknowledge that terms such as “homosexual” and “homosexuality” are considered outdated and may carry pejorative connotations in many Western-based professional and clinical contexts. However, in this manuscript, we use these terms to refer to individuals who are attracted to others of the same gender or sex assigned at birth, in alignment with the direct translation of the commonly used Chinese term “同性恋.” Within the Chinese LGBTQ+ community, this term does not typically carry the same negative connotations. We retain this terminology to preserve linguistic coherence and cultural relevance in the context of this study.
