# Supplementary material for: Does it mean more positive or more negative? A study on central attitudes toward homosexuality
Source: Front Psychol. 2025 Sep 25;16:1635208. doi: 10.3389/fpsyg.2025.1635208 (PMC12507842; doi:10.3389/fpsyg.2025.1635208)
Supplement: Supplementary file 1 [file Table_1.DOCX]

Supplementary Material

# Supplementary Study Materials

## Importance Questionnaire for Inferring Positive Attitudes

*Hello, this survey aims to assess how you* ***infer others’ attitudes toward homosexuality****; it is not an assessment of your own attitudes toward homosexuality. In your opinion, when a person exhibits the following behaviors or viewpoints, which statements best help you determine that they hold* ***a positive attitude toward homosexuality****? Please select six statements from the 12 listed below that you believe indicate the most positive attitudes toward homosexuality.* ***You must select exactly six—no more, no fewer.***

□Respondent: I don’t believe homosexual individuals should be condemned

□Respondent: I don’t believe homosexuality is a sin

□Respondent: I think homosexual individuals should have equal employment opportunities

□Respondent: I think homosexual individuals should be treated fairly in society

□Respondent: I don’t believe homosexuality is an inferior form of sexuality

□Respondent: I don’t believe homosexuality is immoral

□Respondent: I can get along with homosexual individuals comfortably

□Respondent: I can accept my relatives being homosexual

□Respondent: I believe both homosexual and heterosexual individuals can live happy lives

□Respondent: I am willing to be friends with homosexual individuals

□Respondent: I believe that asking homosexual individuals to “remain low-key and avoid being conspicuous” is a form of discrimination

□Respondent: I believe that homosexual individuals should not have to conceal their sexual orientation, living authentically is better for them

## Importance Questionnaire for Inferring Negative Attitudes

*Hello, this survey aims to assess how you* ***infer others’ attitudes toward homosexuality****; it is not an assessment of your own attitudes toward homosexuality. In your opinion, when a person exhibits the following behaviors or viewpoints, which statements best help you determine that they hold* ***a negative attitude toward homosexuality****? Please select six statements from the 12 listed below that you believe indicate the most negative attitudes toward homosexuality.* ***You must select exactly six—no more, no fewer.***

□Respondent: I believe homosexual individuals should be condemned

□Respondent: I believe homosexuality is a sin

□Respondent: I don’t think homosexual individuals should have equal employment opportunities

□Respondent: I don’t think homosexual individuals should be treated fairly in society

□Respondent: I believe homosexuality is an inferior form of sexuality

□Respondent: I believe homosexuality is immoral

□Respondent: I cannot get along with homosexual individuals comfortably

□Respondent: I cannot accept my relatives being homosexual

□Respondent: I believe if homosexual individuals could become heterosexual, they would be happier

□Respondent: I try to avoid being friends with homosexual individuals

□Respondent: I believe if homosexual individuals want to be respected, they should remain low-key and avoid being conspicuous

□Respondent: I believe it is better for homosexual individuals to conceal their sexual orientation

## Representativeness Questionnaire for Inferring Positive Attitudes

*Hello, this survey aims to assess how you* ***infer others’ attitudes toward homosexuality****; it is not an assessment of your own attitudes toward homosexuality. Based on your personal experiences and perspectives, please evaluate the extent to which each of the following behaviors or viewpoints represents a positive or negative attitude toward homosexuality. You will rate each item* ***on a scale from −5 to +5****, where positive scores indicate a positive attitude, negative scores indicate a negative attitude, and a score of 0 means it is difficult to determine. The larger the absolute value, the stronger the perceived positivity or negativity.*

□Respondent: I don’t believe homosexual individuals should be condemned

□Respondent: I don’t believe homosexuality is a sin

□Respondent: I think homosexual individuals should have equal employment opportunities

□Respondent: Please confirm that you understand this survey is not asking about your personal attitudes, but rather about your inference of others’ attitudes, and select (-3)

□Respondent: I think homosexual individuals should be treated fairly in society

□Respondent: I don’t believe homosexuality is an inferior form of sexuality

□Respondent: I don’t believe homosexuality is immoral

□Respondent: I can get along with homosexual individuals comfortably

□Respondent: I can accept my relatives being homosexual

□Respondent: I believe both homosexual and heterosexual individuals can live happy lives

□Respondent: I am willing to be friends with homosexual individuals

□Respondent: I believe that asking homosexual individuals to “remain low-key and avoid being conspicuous” is a form of discrimination

□Respondent: I believe that homosexual individuals should not have to conceal their sexual orientation, living authentically is better for them

## Representativeness Questionnaire for Inferring Negative Attitudes

*Hello, this survey aims to assess how you* ***infer others’ attitudes toward homosexuality****; it is not an assessment of your own attitudes toward homosexuality. Based on your personal experiences and perspectives, please evaluate the extent to which each of the following behaviors or viewpoints represents a positive or negative attitude toward homosexuality. You will rate each item* ***on a scale from −5 to +5****, where positive scores indicate a positive attitude, negative scores indicate a negative attitude, and a score of 0 means it is difficult to determine. The larger the absolute value, the stronger the perceived positivity or negativity.*

□Respondent: I believe homosexual individuals should be condemned

□Respondent: I believe homosexuality is a sin

□Respondent: I don’t think homosexual individuals should have equal employment opportunities

□Respondent: I don’t think homosexual individuals should be treated fairly in society

□Respondent: I believe homosexuality is an inferior form of sexuality

□Respondent: Please confirm that you understand this survey is not asking about your personal attitudes, but rather about your inference of others’ attitudes, and select (-3)

□Respondent: I believe homosexuality is immoral

□Respondent: I cannot get along with homosexual individuals comfortably

□Respondent: I cannot accept my relatives being homosexual

□Respondent: I believe if homosexual individuals could become heterosexual, they would be happier

□Respondent: I try to avoid being friends with homosexual individuals

□Respondent: I believe if homosexual individuals want to be respected, they should remain low-key and avoid being conspicuous

□Respondent: I believe it is better for homosexual individuals to conceal their sexual orientation
